# MOF-derived biochar composites for enhanced high performance photocatalytic degradation of tetracycline hydrochloride†

**DOI:** 10.1039/d2ra05819g

**Published:** 2022-11-08

**Authors:** Zhiwei Liu, Yi Li, Chen Li, Kunyapat Thummavichai, Chen Feng, Zhen Li, Song Liu, Shenghua Zhang, Nannan Wang, Yanqiu Zhu

**Affiliations:** Guangxi Institute Fullerene Technology (GIFT), Key Laboratory of New Processing Technology for Nonferrous Metals and Materials, Ministry of Education, School of Resources, Environment and Materials. Guangxi University Nanning 530004 China shzhang@gxu.edu.cn wangnannan@gxu.edu.cn; College of Engineering, Department of Mathematics and Physical Sciences, University of Exeter Exeter EX4 4QF UK; Faculty of Engineering and Environment, Northumbria University Newcastle Upon Tyne NE1 8ST UK

## Abstract

Biochar reinforced advanced nanocomposites are of interest to a wide circle of researchers. Herein, we describe a novel MOF-derived reinforced cow dung biochar composite, which was prepared by a one-step hydrothermal method to form the MOF MIL-125(Ti) onto a nitrogen and sulfur co-doped bio-carbon (NSCDBC). The UV-vis diffuse reflectance spectrum of NSCDBC/MIL-125(Ti) exhibits an extension of light absorption in the visible region (360–800 nm), indicating its higher visible light capture capacity relative to pure MIL-125(Ti). The photocatalytic activity results show that all the NSCDBC/MIL-125(Ti) composite samples, namely NSCM-5, NSCM-10, NSCM-20 and NSCM-30 display good performance in the removal of tetracycline hydrochloride compared to pure MIL-125(Ti). Among them, NSCM-20 exhibits the highest catalytic activity with a removal rate of 94.62%, which is attributed to the excellent adsorption ability of NSCDBC and the ability to inhibit the complexation of photogenerated electron–hole pairs. Photoluminescence verifies that the loading of biochar successfully enhances the separation of photogenerated electron–hole pairs. Subsequently, the active species in the photocatalytic process are identified by using electron spin resonance spin-trap techniques and free radical trapping experiments. Finally, the possible reaction mechanism for the photocatalytic process is revealed. These results confirm that NSCDBC/MIL-125(Ti) is a potentially low-cost, green photocatalyst for water quality improvement.

## Abstract 2

Schematic diagram of fabricating process of NSCDBC/MIL-125(Ti).

## Graphical abstract

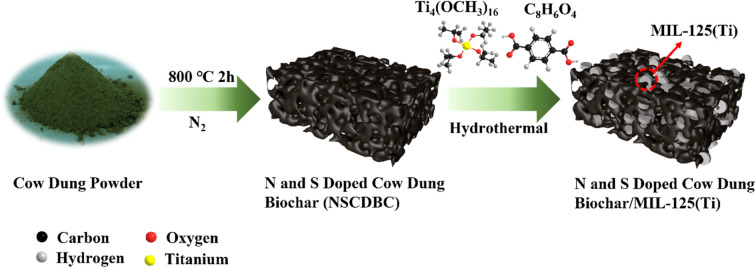

## 1 Introduction

The rapid development of modern medicine and graziery generally comes at the expense of the environment. The discharge of wastewater has been a serious threat to the ecosystem, with the most serious impact being pollution with antibiotic wastewater.^1,2^ Among the many antibiotics, tetracycline hydrochloride (TC) is the most widely used in animal husbandry and medical treatments.^3,4^ TC tends to have a stable structure and a long half-life, and thus cannot be fully absorbed by organisms, resulting in its widespread presence in surface water, coastal environments, soil, groundwater and even drinking water.^5^ TC will induce antibiotic-resistant pathogens when exposed to drinking water for a long time,^6^ which can make the existing drug treatments less effective. Due to its chemical stability, TC is difficult to degrade by conventional treatments.^7^ Therefore, it is essential to find effective methods to degrade tetracycline contaminants. Various techniques have been reported for the elimination of TC from water, such as adsorption,^8^ biodegradation,^9^ electrolysis,^10^ advanced oxidation^11^ and photocatalysis.^12,13^ Among them, photocatalysis is considered as the most promising approach because of its high efficiency, low cost, non-toxicity and environmental friendliness.^14^

There exist a number of disadvantages, like large forbidden bandwidth, inability to make full use of sunlight, and high photo-generated electron–hole complexation rate, for the Conventional semiconductor materials, such as TiO_2_,^15^ g-C_3_N_4_ ref. 16 and ZnO,^17^ which limit their further development and sequence applications. As a rapidly developing coordination polymer in recent years, metal–organic frameworks (MOFs) possess the advantages of tunable chemical properties, regular crystal structure, large specific surface area and high porosity.^18–20^ Therefore, they have gained wide attention in the fields of catalysis,^21,22^ adsorption^23,24^ and light capture.^25,26^ Among many MOFs, MIL-125(Ti) is widely considered as a common photocatalyst due to its easy preparation, high porosity and crystal stability.^27^ However, its higher band-gap of 3.6 eV makes it active only in the UV region.^28^ It is expected to improve its absorption in the visible part by compounding carbon-based materials.

Carbon-based materials have been used to enhance photocatalytic performance because of their excellent charge carrier mobility, good electrical and thermal conductivity,^29^ such as reduced graphene oxide^30^ and carbon nanotubes.^31^ However, carbon nanotubes and graphene have limited application in practical wastewater treatment due to their high cost and complex process.^32^ Compared with other carbon-based materials, BC has the advantages of a wide range of raw materials, simple treatment conditions, and has a good prospect in wastewater treatment.^33^

Biochar (BC) is obtained from the pyrolysis of biological waste such as agricultural residues, food scraps and woody waste under anaerobic conditions.^34^ BC equips the advantages of good strong adsorption ability, various surface functional groups and electrical conductivity, and has become an excellent substrate for enhanced photocatalytic performance.^35,36^ For example, pyrolytic char (PC) is used as the carrier and TiO_2_ is loaded *in situ* to form PC/TiO_2_. Compared with pure TiO_2_, the maximum adsorption capacity of the nano-composite catalyst PC/TiO_2_ for phenol range from 24.7 mg g^−1^ to 41.2 mg g^−1^. PC acts as the receiver for photoelectrons allowing PC/TiO_2_ to exhibit enhanced phenol removal capabilities.^37^ Chen *et al.*^38^*in situ* synthesized a new composite material PbMoO_4_@BC, and the study show that the catalyst with 1 : 4 ratio of PbMoO_4_ to BC showed the best photocatalytic performance, with a substrate concentration of 150 mg L^−1^, the removal rate of TC reach 61.0% more than twice PbMoO_4_ (26%).

Cow manure has a large amount of undigested cellulose and lignin, which is a natural source of carbon. Cow dung based BC can be used as an efficient adsorbent for removing toxic inorganic anions, heavy metals, dyes and other environmental pollutants.^39–41^ Cow-down is un-want waste and has limitation on development or applying into various technologies, only can use as a fertilizer *etc.* No one focus on using it. As a major source of solid waste from the cattle industry,^42^ cattle manure has a high risk of pathogen transmission and infection, and its disposal is considered as a huge challenge for the livestock and farming industry.^43^ Currently, the BC from cattle down is considered to be an effective pollutant treatment strategy.^44^

In this work, for the first time, a porous NSCDBC with low cost, environmentally friendly and excellent adsorption properties was prepared by co-doping of N and S with l-cysteine hydrochloride using cow dung as a carbon source. The NSCDBC/MIL-125(Ti) photocatalytic composite was synthesized *in situ* by a one-step hydrothermal method. After various characterizations of the catalyst, their photocatalytic property was determined by photodegradation of TC. Finally, ESR and free radical trapping experiments are conducted to identify the active species and to explore the pivotal role of NSCDBC in the photocatalytic process.

## 2 Experimental

### 2.1 Chemical reagents

Titanium isopropoxide (Ti_4_(OCH_3_)_16_, Shanghai Macklin Biochemical Co., Ltd), 1,4-benzenedicarboxylic acid (H_2_BDC; C_8_H_6_O_4_, Shanghai Macklin Biochemical Co., Ltd), methanol (CH_3_OH, Shanghai Aladdin Biochemical Technology Co., Ltd), *N*,*N*-dimethylformamide (DMF; (CH_3_)_2_NCHO, Guandong Guanghua Sci-Tech Co., Ltd), sodium bicarbonate (NaHCO_3_, Guandong Guanghua Sci-Tech Co., Ltd), l-semicarbazone hydrochloride (C_3_H_7_NO_2_S–HCl–H_2_O, Sinopharm Chemical Reagent Co., Ltd), anhydrous ethanol (CH_3_CH_2_OH, Guandong Guanghua Sci-Tech Co., Ltd), l-ascorbic acid (l-AA; C_6_H_8_O_6_, Damao Chemical Reagent Factory), 2-methyl-2-propanol (IPA; (CH_3_)_3_COH, Guandong Guanghua Sci-Tech Co., Ltd), disodium ethylenediaminetetraacetate (EDTA-2Na; C_10_H_14_N_2_Na_2_O_8_, Shanghai Macklin Biochemical Co., Ltd) and tetracycline hydrochloride (TC; C_22_H_24_N_2_O_8_, Shanghai Aladdin Biochemical Technology Co., Ltd). All reagents and solvents were analytically pure and not further purified before use.

### 2.2 Preparation of photocatalysts

#### 2.2.1 Synthesis of NSCDBC

Firstly, 2 g cow dung was added to 80 mL deionized water and stirred at 25 °C for 30 min, then 3 g of NaHCO_3_ and 1.270 g of l-cysteine hydrochloride, respectively, were added. The mixed solution was stirred continuously at a constant temperature of 80 °C for 4 h and finally put it in the oven for another 48 h at 60 °C. Bio carbon was ramped up to 800 °C at a rate of 5 °C min^−1^ under N_2_ atmosphere for 2 h. After heat treatment, the sample was washed with ethanol and deionized water to neutral respectively, and dried at 80 °C to obtain NSCDBC.

#### 2.2.2 Preparation of NSCDBC/MIL-125(Ti) composite catalyst

**Fig. 1:**
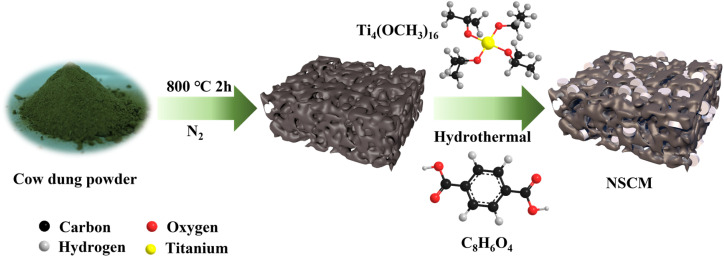
Simplified flow chart for the preparation of NSCDBC/MIL-125(Ti).

Firstly, a set amount of NSCDBC was added to 2 mL anhydrous methanol and 18 mL DMF, and stirred vigorously for 30 min. Then, 1 g H_2_BDC and 0.568 mL Ti_4_(OCH_3_)_16_ were added under stirring, then continuously sonicated for another 20 min. The homogeneous solution was transferred to an autoclave lined with Teflon and held at 150 °C for 18 h. After cooling to room temperature, the solution was centrifuged at 8000 rpm min^−1^ for 5 min and washed with ethanol and DMF, respectively. The sample was dried under vacuum at 80 °C for 12 h to obtain NSCDBC/MIL-125(Ti). The composite catalysts with 5%, 10%, 20% and 30% of biomass carbon content were named NSCM-5, NSCM-10, NSCM-20 and NSCM-30, respectively. The pure MIL-125(Ti) was prepared by the same hydrothermal method as above (Fig. 1).

### 2.3 Analytical methods

The crystal pattern of MIL-125(Ti), NSCDBC and NSCDBC/MIL-125(Ti) composites were analyzed by X-ray diffractometer (XRD, Rigaku D/MAX 2500 V, Rigaku Corporation), working under 40 kV accelerating voltage with Cu Kα1 radiation (*λ* = 1.54056 Å) characterized by recording 2*θ* in the range of 5° to 80°. Scanning electron microscopy (SEM, Sigma 300, Carl Zeiss) and transmission electron microscopy (TEM, Tecnai F20, FEI) were used to study the microstructure and morphology of the as-prepared photocatalysts. A surface area analyzer (TriStar II 3020, Micromeritics) was used to analyze the specific surface area and pore volume of the synthesized photocatalysts. UV-vis diffuse reflectance spectra (DRS) was acquired on a Lambda 365 spectrophotometer (PerkinElmer). Fourier transform infrared (FT-IR) spectra of photocatalysts were collected by Fourier transform infrared spectrometer (Nicolet iS50, Thermo Fisher Scientific). X-ray photoelectron spectroscopy (XPS) of composites was performed on ESCALAB 250XI (Thermo Fisher) using Al *K*α source. The UV spectrometer (Lambda 365, PerkinElmer) was used to measure the concentration of TC in this study. Total organic carbon (TOC) was obtained by the Shimadzu TOC-L CPH analyzer. The photoluminescence (PL) spectra of the composites were acquired by the FL3C-111 TCSPC spectrometer. Thermogravimetry Analysis (TGA) spectra of the composites were acquired by PerkinElmer TGA4000.

### 2.4 Photocatalytic experiment

The photocatalytic performance of the as-prepared NSCDBC/MIL-125(Ti) samples was evaluated by degrading TC under light conditions at an initial concentration of 40 mg L^−1^. The light source was a 300 W xenon lamp. In this procedure, 20 mg of as-prepared sample was added into 100 mL contaminant solution and stirred in the darkness condition for 60 min ensure an adsorption–desorption equilibrium between the catalyst and the contaminant. Then, the suspension is illuminated and a 4 mL sample is taken every 10 minutes and centrifuged to remove the catalyst particles. After completion of irradiation, the NSCM-20 pellets were recovered by centrifugation, washed several times with ultrapure water, and then dried. After that, the stability of TC was evaluated by direct photocatalytic degradation of TC using NSCM-20. The catalytic efficiency is reflected by the variation of the TC concentration determined by the UV spectrophotometer studied at the maximum absorption wavelength of 360 nm.

## 3 Results and discussion

### 3.1 Morphology characterizations

**Fig. 2:**
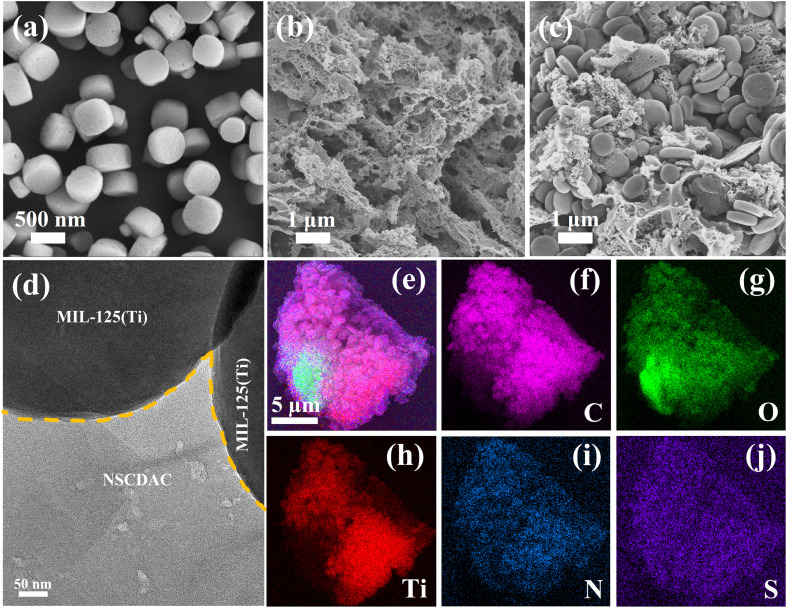
SEM images of as-prepared samples: (a) pure MIL-125(Ti); (b) NSCDCA; (c) NSCM-20, TEM images of (d) NSCM-20, (e–j) SEM-EDS mapping images of NSCM-20.

The surface morphologies of NSCDBC, pure MIL-125(Ti) and NSCM-20 were analyzed by SEM and TEM. This can be seen in Fig. 2a and b, NSCDBC microspores are formed during the pyrolysis of cow dung. The MIL-125(Ti) material performs a pie-shaped structure with a radius of 0.3–0.5 μm, a thickness of about 0.25 μm, and a relatively smooth surface. For NSCM-20 composite sample, it can be clearly seen that MIL-125(Ti) is attached to the surface of NSCDBC (Fig. 2c). Further results from TEM of the NSCDBC/MIL-125(Ti) composite are given. NSCDBC shows that MIL-125(Ti) is grown *in situ* on NSCDBC with a tight bond between them (Fig. 2d). In addition, SEM-EDS mapping results also confirms that the C, O, Ti, N and S elements are uniformly distributed in one NSCDBC, indicating that MIL-125(Ti) particles are uniformly attached to the NSCDBC surface (Fig. 2e–j).

### 3.2 Composition and structure characterizations

**Fig. 3:**
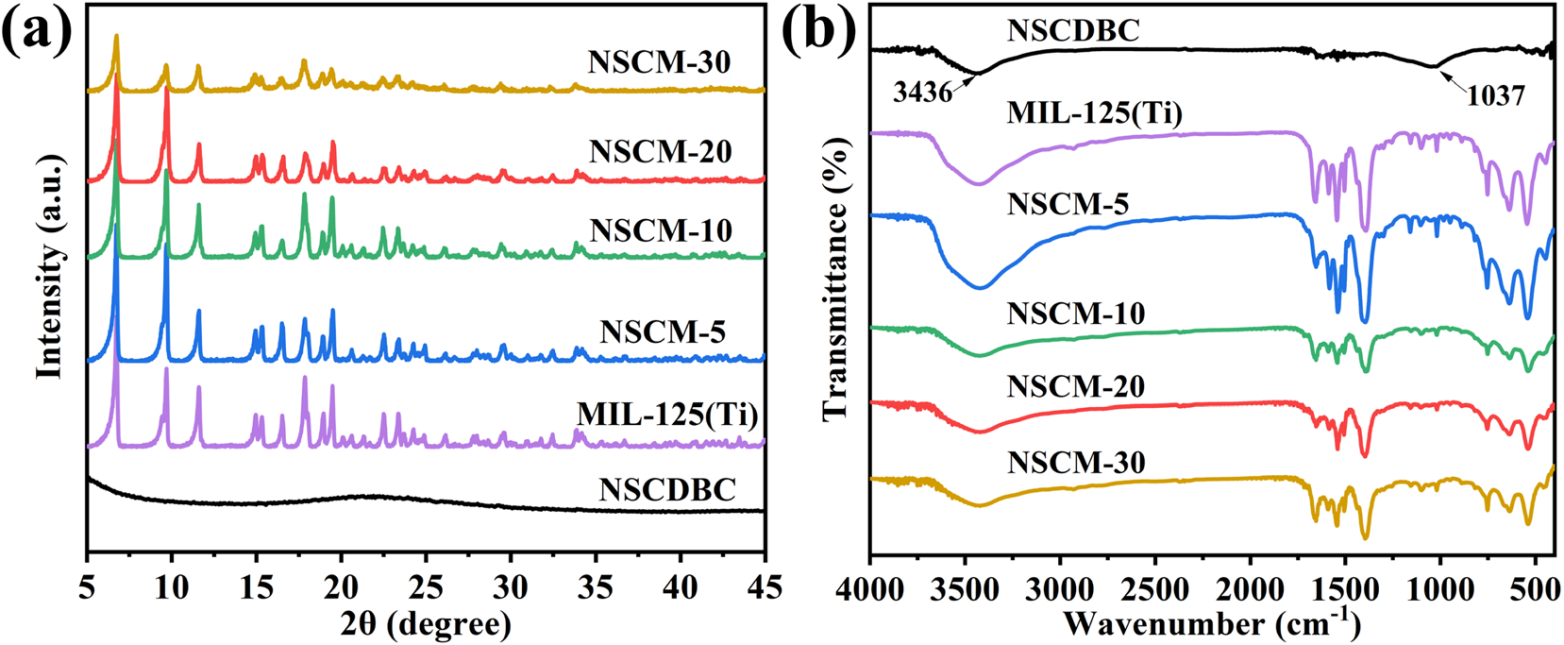
(a) XRD patterns of MIL-125(Ti), NSCDBC and NSCDBC/MIL-125(Ti); (b) FT-IR spectra of NSCDBC, MIL-125(Ti) and NSCDBC/MIL-125(Ti).

The phase structures of MIL-125(Ti) and composites by X-ray diffractometry (XRD) are shown in Fig. 3a. For NSCDBC, the broad peaks at 2*θ* = 23° and 43° are designated as characteristic peaks of graphitic carbon. For pure MIL-125(Ti), the XRD diffraction peaks at 6.9°, 9.8° and 11.7° correspond to the (101), (200) and (221) planes of the MIL-125(Ti) crystal, respectively, which are in full agreement with those reports previously.^45,46^ It indicates that pure MIL-125(Ti) is successfully prepared. No typical diffraction peaks change can be found in the NSCDBC/MIL-125(Ti) composite sample, but a change in intensity. This result indicates that MIL-125(Ti) crystals can be formed perfectly in the presence of biomass carbon. It is noteworthy that no graphitic carbon peak is observed in the XRD pattern of NSCDBC/MIL-125(Ti) sample, which is due to NSCDBC is amorphous and the content is low. This clearly demonstrates the successful preparation of NSCDBC/MIL-125(Ti) composites.

FT-IR is used to study the functional groups of composite materials. As shown in Fig. 3b, for NSCDBC samples, the broad peak at 3380–3700 cm^−1^ is caused by the adsorption of water molecules.^47^ The absorption peak at 1037 cm^−1^ corresponds to the symmetric stretching vibration of C–O–C. For MIL-125(Ti), the broad peak at 3424 cm^−1^ is caused by solvent molecules adsorbed in MIL-125(Ti). The broad peaks at 1655 and 1400 cm^−1^ are attributed to the vibrational stretching of O–C–O in the framework, which also confirms the presence of terephthalate linker in MIL-125(Ti). There is Characteristic absorption peak located at 1013 and 740 cm^−1^, which belong to benzene rings. The bands between 400 and 800 cm^−1^ origin from O–Ti–O vibrations.^48^ NSCDBC/MIL-125(Ti) composites show characteristic peaks similar to those of MIL-125(Ti), and when the ratio of NSCDBC/MIL-125(Ti) is changed, a change in the intensity of the above-infrared absorption bonds can be observed, indicating the existence of interaction forces between MIL-125(Ti) and NSCDBC. Therefore, NSCDBC/MIL-125(Ti) composites are successfully prepared.

**Fig. 4:**
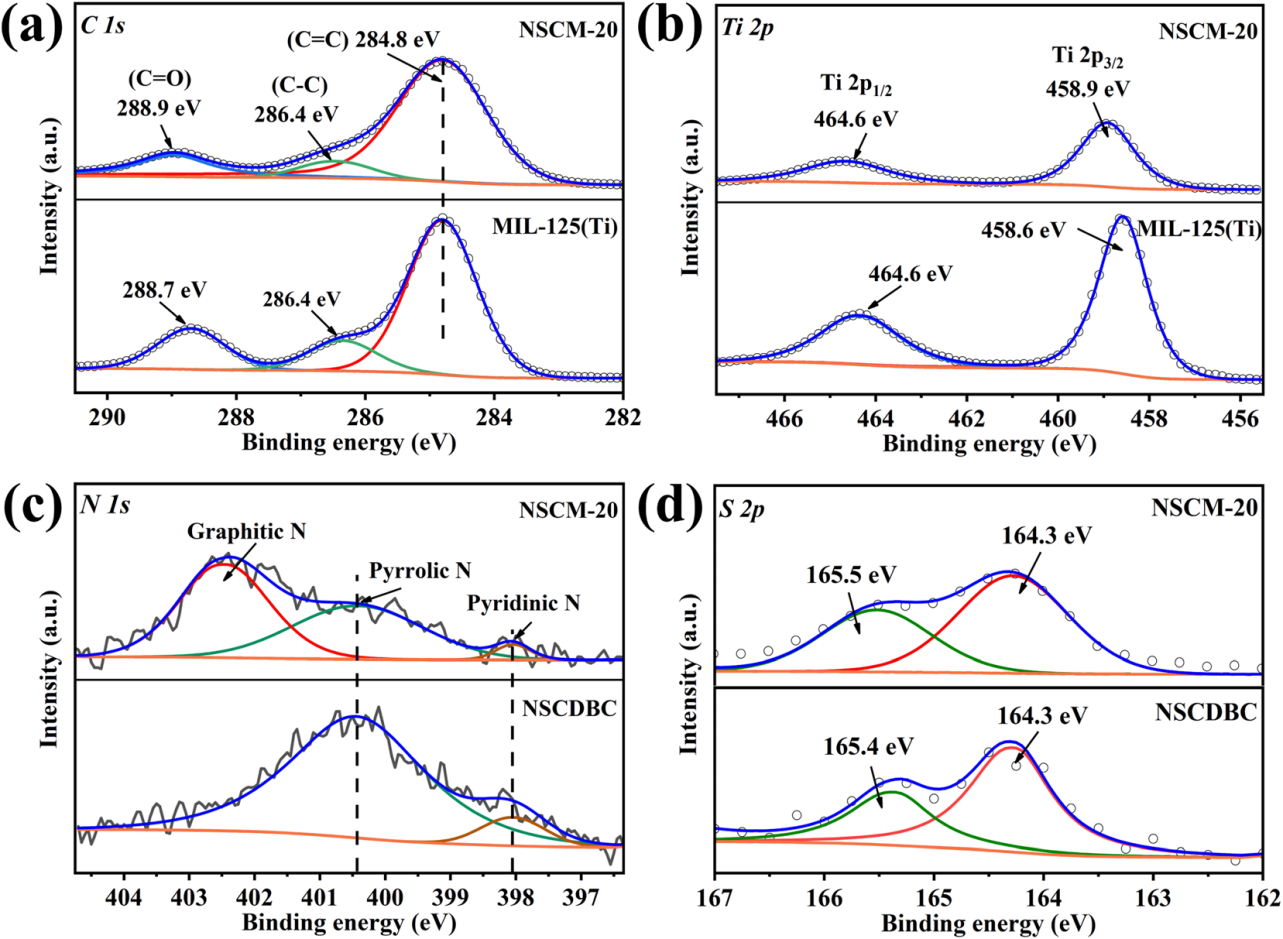
XPS spectra of NSCDBC, MIL-125(Ti) and NSCM-20: (a) C 1s, (b) Ti 2p, (c) N 1s, (d) S 2p.

The elemental valence surface chemistry and surface chemistry of the composites are further analyzed by XPS, the results of the high-resolution XPS spectra of C 1s, N 1s, S 2p and Ti 2p in the NSCDBC, MIL-125(Ti) and NSCM-20 are shown in Fig. 4. Three peaks are found at 284.8, 286.38 and 288.7 eV in the C 1s spectra of pure MIL-125(Ti) (Fig. 4a), which comes from the sp^2^ hybridization of carbon (C=C),^49^ C–C and carboxylate group on H_2_BDC (O–C=O). The C 1s spectrum of NSCM-20 shows a shift of the carbon-oxygen double bond toward the high binding energy of about 0.26 eV. The high-resolution S 2p peaks of both NSCDBC and NSCM-20 are fitted to two components located around 164 and 165 eV in Fig. 4d, For NSCDBC, the two peaks correspond to the 2p_3/2_ and 2p_1/2_ orbitals of S, respectively, indicating the incorporation of S into the carbon framework.^50^ Note that the S 2p_3/2_ charge is shifted in the sample of NSCM-20. Fig. 4c shows the N 1s spectrum of NSCDBC and NSCM-20. The N 1s spectrum of NACDBC can be decomposed into two peaks, which attribute to pyrrolic-like nitrogen (400.43 eV) and pyridine-like nitrogen (398.04 eV),^32,51^ while the N and S contents are 1.9% and 0.56%, respectively. The above results indicate the successful binding of N and S substances into the carbon framework and creates a synergistic effect.

In addition to the characteristic peaks of pyrrole nitrogen and pyridine nitrogen in the NSCM-20 spectrum, a new characteristic peak appears at 402.45 eV, which corresponds to graphitic nitrogen. This is meaningful for improving the catalytic performance of the oxygen reduction reaction (ORR).^52^Fig. 4b shows the comparison of Ti 2p spectra between NSCM-20 and MIL-125(Ti). It can be seen that two peaks appear at 458.57 and 464.31 eV from the Ti 2p_3/2_ and 2p_1/2_ orbitals, respectively, indicating that titanium is present in the titanium cluster in the IV-valent form.^53,54^ Moreover, the characteristic peaks of Ti shift toward higher binding energies after NSCDBC loading, which is attributed to the possible formation of Ti-OC bond inside the composite structure.^55^ The above results show that well-bonded NSCDBC/MIL-125(Ti) composite material is successfully synthesised.

**Fig. 5:**
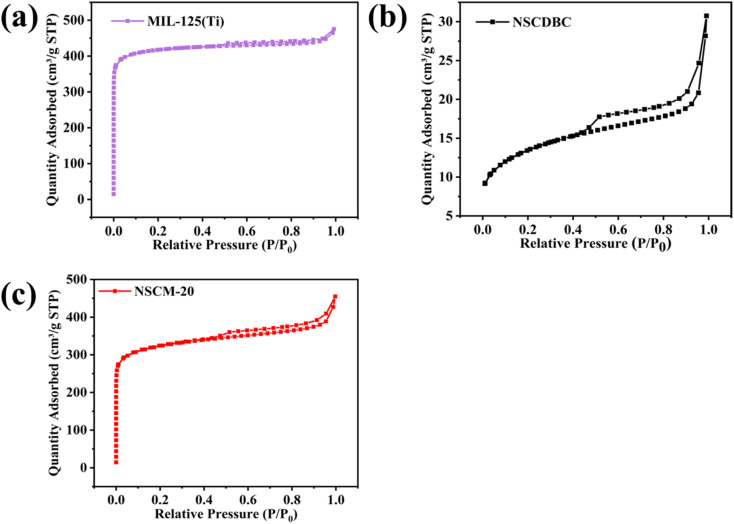
Nitrogen absorption–desorption isotherms of (a) pure MIL-125(Ti), (b) NSCDBC and (c) NSCM-20.

There is characterization of specific surface area, pore size distribution and total pore volume of composites by means of BET specific surface area analysis. As shown in Fig. 5a, pure MIL-125(Ti) exhibits a type I adsorption isotherm, indicating a typical microporous structure. A pore size distribution of about 0.48 nm is estimated by using the Horvath-Kawazoe method (Fig. S1†).^56^ However, a very small hysteresis loop can be observed in the figure (*P*/*P*_0_, 0.47–1.0), the formation of which is related to capillary condensation, indicating that a certain mesoporous/macropore structure is also present in the MIL-125(Ti) structure. The presence of a small amount of mesoporous structure can also be observed by the Barrett–Joyner–Halenda method (Fig. S2†). On the other hand, pure NSCDBC presents as a type IV adsorption isotherm with the presence of mesopores (2–50 nm) at Fig. 5b and S3.† In addition, the hysteresis return line of pure NSCDBC is H3 type, which implies the presence of slit-like pores and capillary agglomerates in mesopores.^57^Fig. 5c shows NSCM-20 exhibits mixed type I and IV adsorption isotherms with a large hysteresis return line, indicating the presence of micro-and mesopores. In the photocatalytic process, the presence of a certain number of mesoporous structures facilitates the adsorption of pollutants by the catalyst and improves the photocatalytic reaction efficiency.

The specific pore volume and surface area of these three catalysts are shown in Table 1. The specific surface area and pore volume of MIL-125(Ti) are 1925.92 m^2^ g^−1^ and 0.566 cm^3^ g^−1^, respectively, and the corresponding values of NSCDBC are 99.80 m^2^ g^−1^ and 0.048 cm^3^ g^−1^, respectively. The specific surface area and pore volume of NSCM-20 are between the values of the two individual substances, corresponding to 1626.94 m^2^ g^−1^ and 0.376 cm^3^ g^−1^. MIL-125(Ti) and NSCM-20 have high microporosity. The larger the specific surface area of the catalyst is, the smaller the microporous pore size will be, and the more adsorption and catalytic sites will possess, which facilitate the removal of the target contaminant, in this case tetracycline hydrochloride.

**Table 1:** Specific surface area and pore volume of the as-prepared samples

Samples	Specific surface area (SSA) (m^2^ g^−1^)	Pore volume (cm^3^ g^−1^)
MIL-125(Ti)	1925.92	0.566
NSCDBC	99.80	0.048
NSCM-20	1626.94	0.376

### 3.3 Optical and electronic properties

**Fig. 6:**
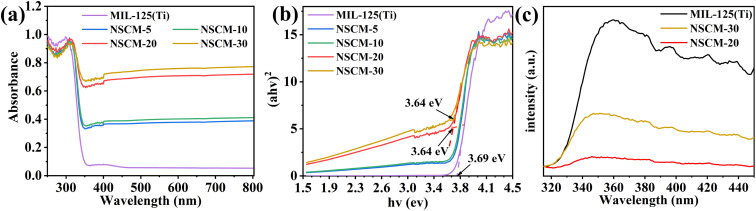
(a) UV-vis absorption spectra of pure MIL-125(Ti) and different NSCDBC/MIL-125(Ti) composite materials, (b) bandgap energy of photocatalysts, (c) PL spectra of MIL-125(Ti), NSCM-20 and NSCM-30.

The light absorption ability and band-gap energy of the catalyst largely determine the activity of the catalyst. As shown in the Fig. 6a, all samples exhibit very good absorption in the UV region (*λ* < 300 nm). The absorption edge of pure MIL-125(Ti) is around 350 nm, and it almost does not absorb visible light at wavelengths greater than 400 nm. After loading NSCDBC, the composite catalysts exhibit a significant absorption enhancement in the visible band, indicating their ability to utilize visible and UV light for photocatalytic processes.

In addition, the band gap of all catalysts are estimated based on the Kubelka-Munk equation.^58^(*F*(*R*_∞_) × *hυ*)^2^ = *B*(*hυ* − *E*_g_)12
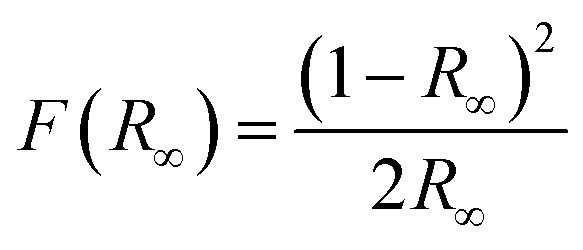
3
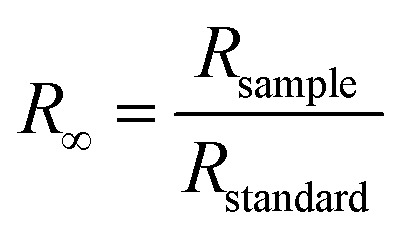
where *R*_∞_ is the reflectance of an infinitely thick specimen; *R*_standard_ is the reflectance of BaSO_4_; *R*_sample_ is the reflectance of catalyst; *h* is the Planck constant; *ν* is the photon's frequency; *B* is a constant; *E*_g_ is the band-gap energy.

Due to modifications, including surface doping, defects, and blocking, an in-band gap state may be introduced, resulting in an inaccurate estimate of *E*_g_. Therefore, the linear fit to the basal peak and the linear fit to the slope below the basal absorption are given, and the transverse coordinate of the intersection point is the band gap estimate (Fig. 6b). The band-gap of pure MIL-125(Ti), NSCM-5, NSCM-10, NSCM-20 and NSCM-30 are 3.69, 3.66, 3.65, 3.64 and 3.64 eV, respectively, indicating that the loading of biomass carbon has no effect on the narrowing of the band-gap energy but promotes the absorption of the visible fraction. The obtained results are in agreement with other literature reports.^37^

As previously mentioned, NSCDBC has the effect of enhancing the separation of photogenerated electron–hole pairs, which results in the excellent photocatalytic performance of NSCDBC/MIL-125(Ti) composites. To prove this conclusion, PL measurements are used to examine the separation efficiency of photogenerated electron–hole pairs in the composites. In general, the weaker intensity of the PL result, the lower recombination of photoexcited charge carriers.^59^ Notably, the emission intensity of NSCM-20 is significantly lower than that of pure MIL-125(Ti), indicating that the photogenerated electron–hole separation is more effective in NSCM-20 (Fig. 6c). We suggest that NSCDBC can indeed act as an effective electron acceptor and hinder the complexation of photogenerated carriers under illumination. However, the electron–hole separation efficiency decreases when the NSCDBC content is higher than 30%, indicating that the excess NSCDBC is not favorable for charge separation. This conclusion is alignment with the impunity of photocatalytic performance tests.

### 3.4 Photocatalytic experiment

#### 3.4.1 Photocatalytic activity

**Fig. 7:**
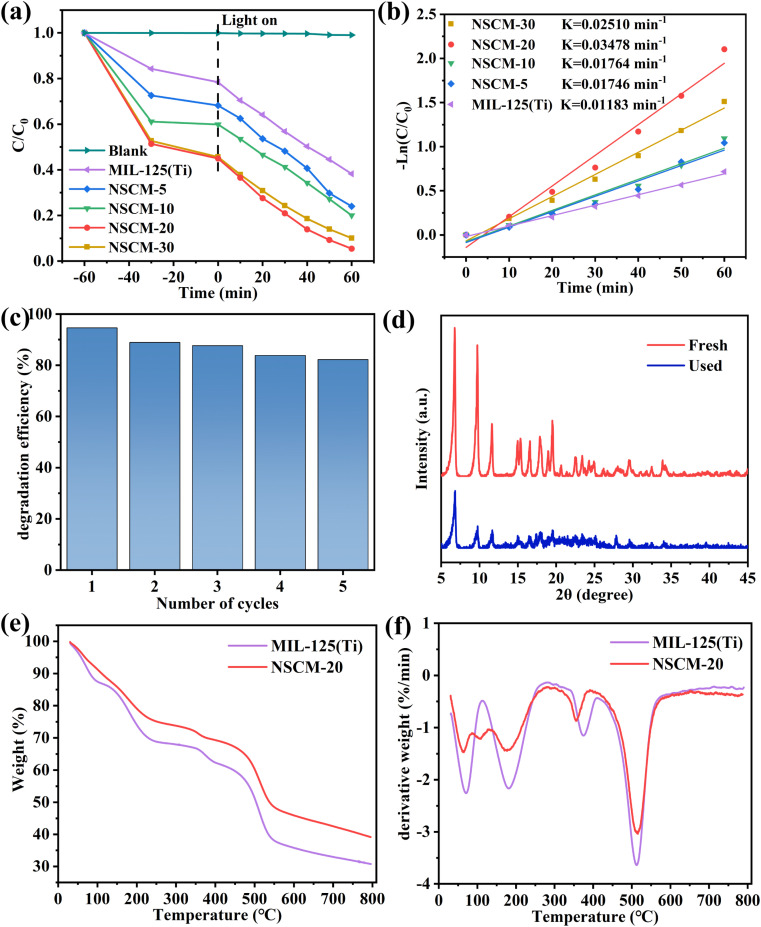
(a) Photocatalytic degradation of TC with different samples under full-spectra light irradiation (*λ* > 365 nm); (b) the corresponding pseudo-first-order kinetic plots; (c) the photocatalytic degradation efficiency of NSCM-20 with 5 cycles; (d) XRD analysis of fresh and used NSCM-20; TGA (e) and DTG (f) of MIL-125(Ti) and NSCM-20.

The above analysis shows that the NSCDBC/MIL-125(Ti) sample has better optical and electronic properties than MIL-125(Ti), which may make for photocatalytic activities of the NSCDBC/MIL-125(Ti) composite. To prove this hypothesis, the photocatalytic activity of the composite catalysts is evaluated by the degradation of antibiotic TC in the full spectrum. As shown in Fig. 7a, the results show that the degradation of TC is negligible in the absence of any catalyst. The addition of NSCDBC promotes the catalytic performance of MIL-125(Ti). The removal efficiency of pure MIL-125(Ti) for TC is only 61.74% after 120 min of light irradiation due to its high band-gap energy and high photogenerated carrier-hole complexation rate. NSCM-5, NSCM-10, NSCM-20 and NSCM-30 composites all exhibit large degradation rates relative to pure MIL-125(Ti), with the removal efficiencies for TC of 75.97%, 79.99%, 94.62% and 90.92%, respectively. The results show that NSCM-20 offers the greatest promise in degrading TC, while the TOC removal reaches to 84.01%, indicating its ability to degrade TC to small molecules and even mineralize it to CO_2_ and H_2_O. In addition to this we also performed the same photocatalytic experiments as before at different pH (Fig. S6†). From the histogram, it shows that the degradation rate of TC under strong acid conditions (pH = 3) slightly decreases, but also reaches 80.12%, and under strong base conditions (pH = 11), the degradation rate reaches 90.51%. This confirms that the composites have good stability and catalytic properties even under strong acid and base conditions.

The excellent degradation performance of NSCM-20 is attributed to the synergistic effect between MIL-125(Ti) and BC. Three main effects of NSCDBC can be summarized as: (i) NSCDBC acts as an effective electron transport channel and acceptor to improve the separation of photogenerated electron–hole pairs, thus positively favoring the photocatalytic oxidation efficiency; (ii) the NSCDBC can promote light absorption as shown by UV-vis spectroscopy analysis; (iii) the abundant functional groups on the surface of NSCDBC have a strong affinity for organic substances thus enabling TC to be enriched on its surface. It is noteworthy that the removal efficiency of NSCM-30 slightly decreases, mainly due to the excess of biochar blocking the light reaching the catalyst surface.

The kinetics of TC photocatalytic degradation is investigated, and the variation of TC concentration with irradiation time for all prepared samples is in accordance with the quasi-level kinetic curve as shown in Fig. 7b. The equation-ln(*C*/*C*_0_) = *k*_app_*t*, where *t*, *C*_0_ and *C* are the reaction time, initial TC concentration (mg L^−1^) and TC concentration at time *t* (mg L^−1^), respectively. *k* represents the apparent pseudo-primary rate constant (min^−1^).^60^ The highest rate constant for NSCM-20 (0.03478 min^−1^) is approximately the same as that for pure MIL-125(Ti) (0.01183 min^−1^), which is consistent with the photocatalytic activity performance.

#### 3.4.2 Catalyst stability

The stability of NSCM-20 was studied by cyclic experiments to assess the stability in long-term applications. As can be seen in Fig. 7c, the degradation rate of TC still reached more than 80% after five consecutive cycles of degradation, indicating the high stability of NSCM-20. Meanwhile, XRD analysis of the catalyst before and after use showed no significant change in the position of the diffraction peaks, confirming the stability of the composite (Fig. 7d). The TGA patterns (Fig. 7e and S5a†) shows that the residual mass percentage of NSCM-20 is 40 wt% higher than that of MIL-125(Ti) when the temperature reaches 800 degrees, and the different composites are also like this. The pyrolysis rate of NSCM-20 is lower than that of MIL-125(Ti) during the temperature increase according to the DTG (Fig. 7f). The same is true for all composite materials (Fig. S5b†). It indicates that the thermal stability of the composite is significantly improved.

#### 3.4.3 Photodegradation mechanism

**Fig. 8:**
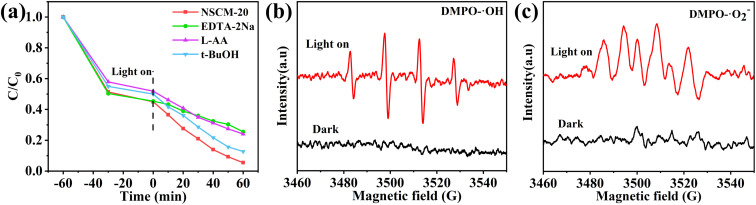
Active species trapping experiment during the photocatalytic degradation of TC over NSCM-20 (a) and DMPO spin-trapping ESR spectra recorded with NSCM-20 under light irradiation (b and c).

Detection of the main active substances in the photocatalytic process by free radical capture experiments. Three different scavengers were added separately during the photodegradation of TC,^61^ including EDTA-2Na (h^+^ scavenger), l-AA (superoxide radical-O^2−^ scavenger) and *t*-BuOH (hydroxyl radical-OH scavenger). As shown in the Fig. 8a, the photocatalytic process is slightly inhibited by the addition of *t*-BuOH, indicating that ˙OH provide the certain influence but does not play a primary role in the photodegradation process. However, the photodegradation is significantly inhibited with the accretion of l-AA, which is only 75.92%, indicating that ˙O^2−^ have an effect on the photodegradation reaction. Meanwhile, the degradation of TC is also significantly suppressed upon the additional EDTA-2Na, indicating that h^+^ significantly contributes in TC photodegradation. Thus, ˙O^2−^, h^+^ and ˙OH all take part in the photodegradation process, and particularly, ˙O^2−^ and h^+^ are the primary active substances in the photocatalytic process.

ERS is used to characterize the active particles produced by the catalyst in aqueous solution. As shown in the Fig. 8b and c, both ˙OH and ˙O^2−^ radicals show negligible characteristic signals under dark conditions. It can be clearly seen that it shows the clear ˙O^2−^ characteristic peak and also detects the signal of ˙OH radicals after illumination. Thus, the ESR results further demonstrate that limited ˙OH radicals come into being in the NSCM-20 system, and the addition of *t*-BuOH has little effect on the removal rate of ˙OH radicals. This is due to the low concentration of ˙OH radicals.^62^

**Fig. 9:**
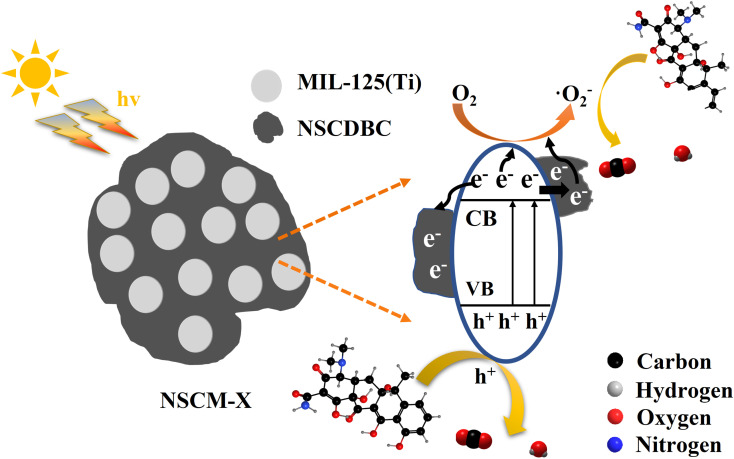
Schematic of the separation and transfer of photogenerated charges in NSCDBC/MIL-125(Ti) system combined with the possible photocatalytic mechanism.

Based on the above experiments and analysis, a reasonable degradation mechanism of NSCM-20 is proposed (Fig. 9). Firstly, biochar adsorbs TC molecules attribute to its high porosity and abundant functional groups (*i.e.* C–O–C, –OH, C–O), which provide more opportunities for contact between the catalyst and the pollutant. When exposed to light, NSCM-20 is photoexcited to generate electrons and transferred to the conduction band while leaving holes in the valence band. Then NSCDBC transfers the electrons and acts as a carrier for the electrons, inhibiting the complexation of electron–hole pairs. Next, the electrons react with oxygen molecules to produce superoxide radicals and due to their lower valence band cannot drive water molecules to form hydroxyl radicals, but instead photogenerated holes are transferred to the catalyst surface to interact with TC molecules^63^ This is consistent with the results of radical trapping experiments and ESR tests. Thus, hydroxyl radicals and photogenerated holes interact together with TC molecules as active substances, and eventually, TC is degraded to CO_2_.

## 4 Conclusion

In this study, value-added utilization of waste biomass has been achieved by converting cow down into modified biomass char. The N,S co-doped biochar derived from cow dung pyrolysis was used to modify MIL-125(Ti) to achieve a cost-effective composite catalyst. The composite material offers great adsorption performance on TC. The effective linkage of biomass char with MIL-125(Ti) was confirmed by SEM, TEM and XPS analysis and they both existed in the pristine state. NSCM-20 exhibits the best photodegradation performance with a degradation efficiency of 94.62% (TOC removal 84.01%), which is higher than that of MIL-125(Ti), indicating that there is a synergistic effect between NSCDBC and MIL-125(Ti). The proper addition of NSCDBC is significantly beneficial to the photocatalytic process. The porous structure and the transfer of photogenerated carriers are the main reasons for the improved photocatalytic performance. In addition, by radical trapping experiments and ESR, photogenerated holes and superoxide radicals are the main oxidizing substances for degrading TC molecules, and a reasonable explanation of the photocatalytic degradation mechanism is made. Therefore, the NSCDBC/MIL-125(Ti) composite catalyst is a new economic and environmentally friendly high-efficiency photocatalyst for the removal of organic pollutants from the aqueous phase.

## Data availability

The raw/processed data required to reproduce these findings cannot be shared at this time as the data also forms part of an ongoing study.

## Author contributions

Zhiwei Liu: writing – original Draft, conceptualization, investigation; Yi Li: formal analysis; Chen Li: data curation; Kunyapat Thummavichai: editing; Chen Feng: methodology; Zhen Li: data curation; Song Liu: validation; Shenghua Zhang: writing – reviewing and editing; Nannan Wang and Yanqiu Zhu: resources, funding acquisition.

## Conflicts of interest

The authors declared that they have no conflicts of interest to this work.

## Supplementary Material

RA-012-D2RA05819G-s001
