# Supplementary material for: MOF-derived biochar composites for enhanced high performance photocatalytic degradation of tetracycline hydrochloride
Source: RSC Adv. 2022 Nov 8;12(49):31900–10. doi: 10.1039/d2ra05819g (PMC9641384; doi:10.1039/d2ra05819g)
Supplement: RA-012-D2RA05819G-s001 [file RA-012-D2RA05819G-s001.pdf]

Electronic Supplementary Material (ESI) for RSC Advances.
This journal is © The Royal Society of Chemistry 2022

Supporting Information

MOF-derived Biochar Composites for Enhanced High Performance Photocatalytic Degradation of Tetracycline Hydrochloride

Zhiwei Liu[a], Yi Li[a], Chen Li[a], Kunyapat Thummavichai[b,c], Chen Feng[a], Zhen Li[a], Song Liu[a], Shenghua Zhang[a]*, Nannan Wang[a]*, Yanqiu Zhu[a,b]

* *Corresponding author. Shenghua Zhang, Nannan Wang*

E-mail address: *shzhang@gxu.edu.cn*, *wangnannan@gxu.edu.cn*

[a] Guangxi Institute Fullerene Technology (GIFT), Key Laboratory of New Processing Technology for Nonferrous Metals and Materials, Ministry of Education, School of Resources, Environment and Materials. Guangxi University, Nanning 530004, China

[b] College of Engineering, Department of Mathematics and Physical Sciences, University of Exeter, Exeter, EX4 4QF, United Kingdom

[c] Faculty of Engineering and Environment, Northumbria University, Newcastle upon Tyne NE1 8ST, United Kingdom

1. Preparation of NSCDBC/MIL-125(Ti) composite catalyst

NSCM-x: Firstly, added 2 mL of anhydrous methanol and 18 mL of DMF to the beaker, then add 25 mg of NSCDBC while stirring, and stirred for 30 min. Then 1 g of H_2BDC was added while stirring and 0.568 mL of $Ti_4(OCH_3)_{16}$ was added after mixing uniformly. Finally sonicated for 20 min to obtain the colloid. After cooling to room temperature, the solution was centrifuged at 8000 rpm·min^{-1} for 5 min using DMF and methanol three times and dried under vacuum at 80 °C for 12 h. The powder obtained was named NSCM-5 according to the content of NSCDBC in the composite. NSCM-10, NSCM-20 and NSCM-30 correspond to 50 mg, 100 mg and 150 mg of NSCDBC addition, respectively.

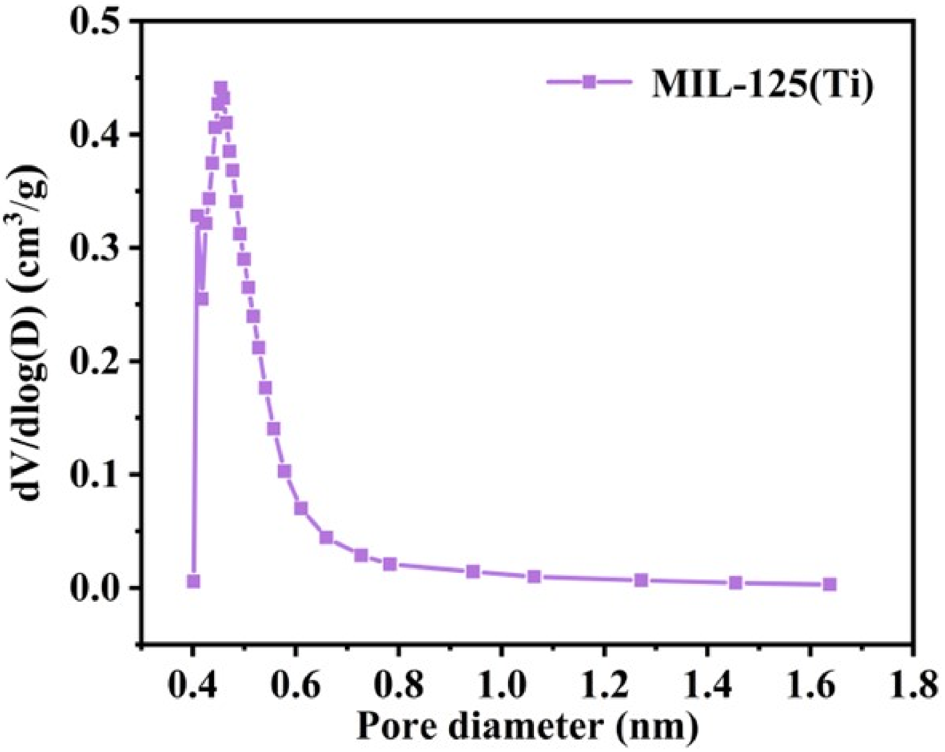

Fig. S1 Pore size distribution of pure MIL-125(Ti) by using the Horvath-Kawazoe method.

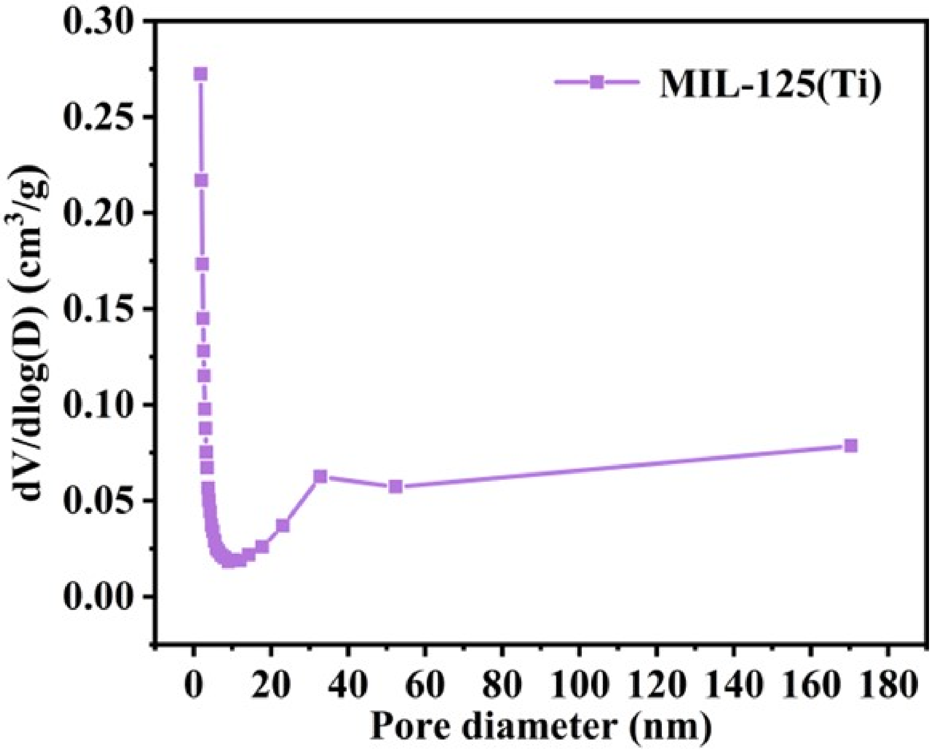

Fig. S2 Pore size distribution of pure MIL-125(Ti) by using the Barrett-Joyner-Halenda method.

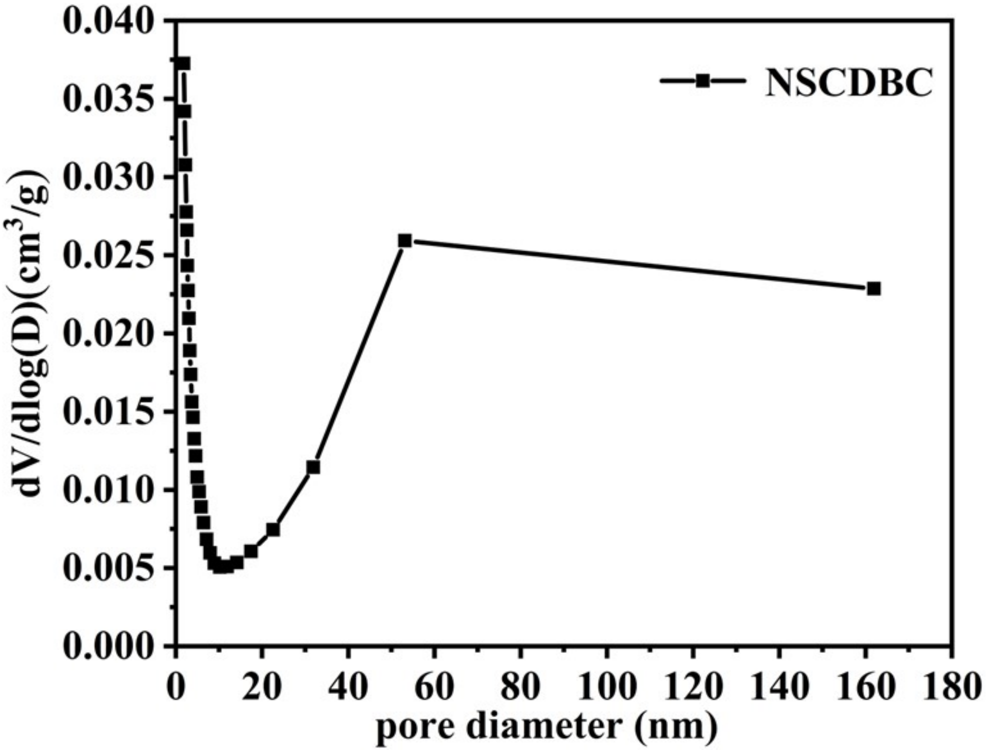

Fig. S3 Pore size distribution of NSCDBC.

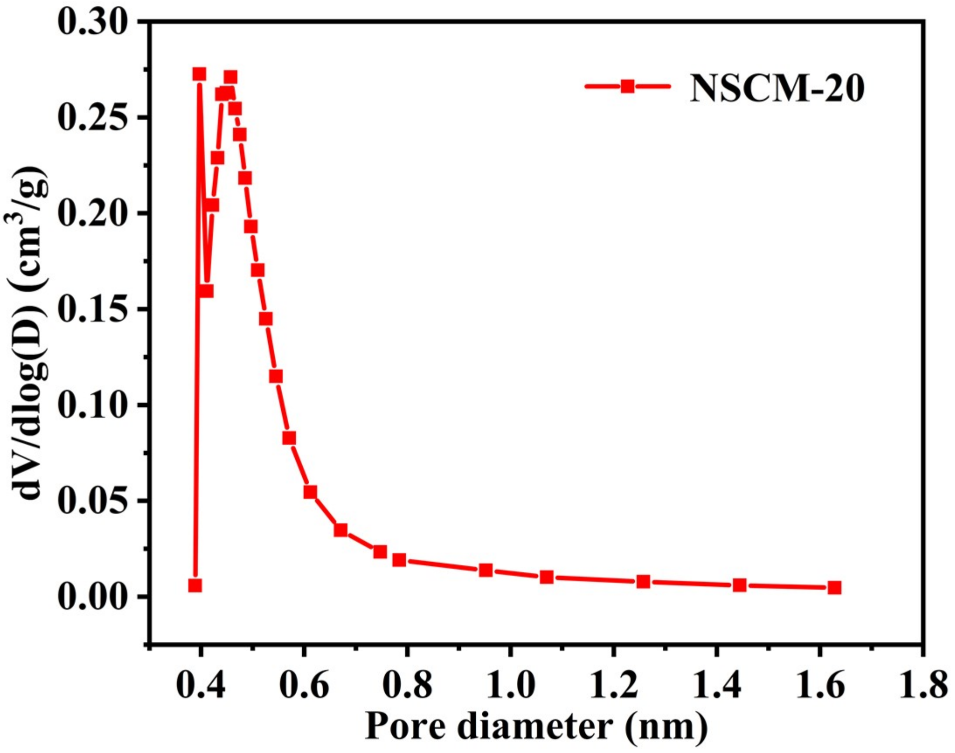

Fig. S4 Pore size distribution of NSCM-20.

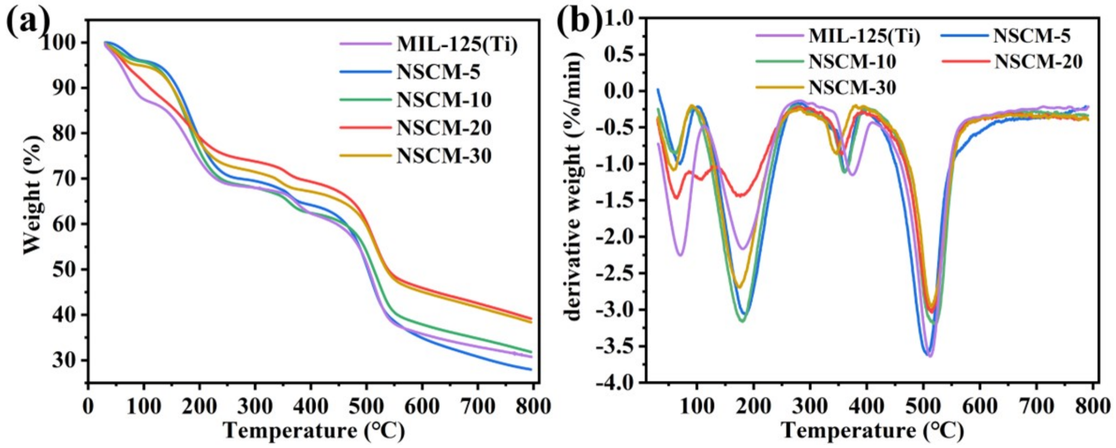

Fig. S5 (a) TGA and (b) DTG patterns of the prepared samples.

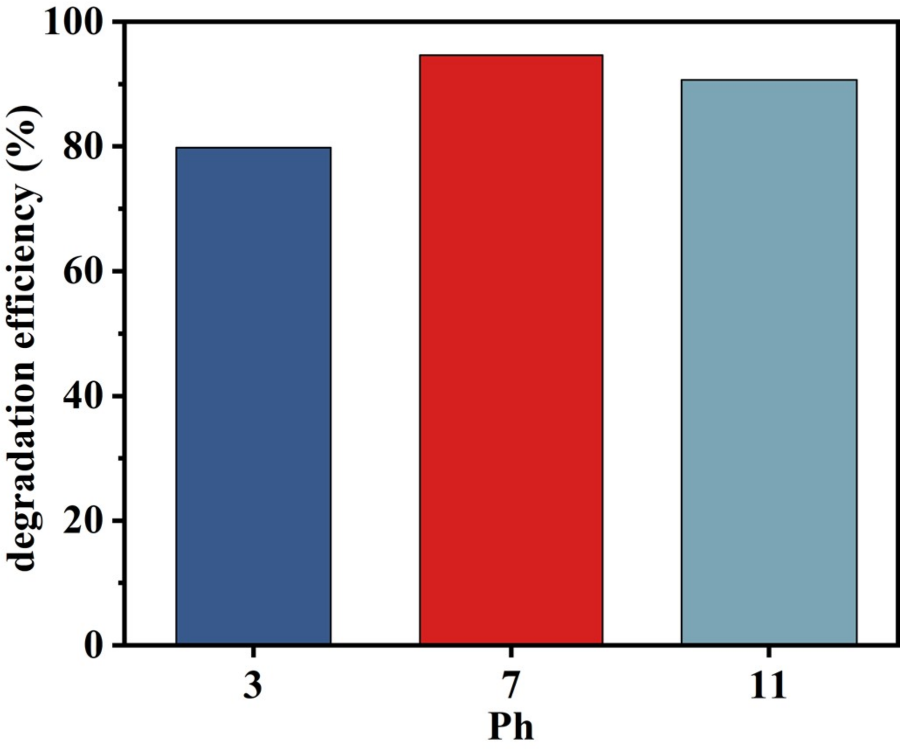

Fig. S6 Degradation rate of TC by NSCM-20 with different Ph.

Table S1. Data of Wastewater Treatment Processes using TiO2/Biochar Systems

**material**	**feedstock**	**pyrolysis temp (°C)**	**pollution**	**initial pollution concentration (mg/L)**	**applied dose (g/L)**	**degradation method**	**removal eficiency (%)**	**ref**
TiO_2/biochar	hemp stem	500	ammonia nitrogen	100	0.03	catalysis (UV)	~99.0	(1)
TiO_2/biochar	Daphnia magna	325	sulfamethoxazole	10	5.00	adsorption + photocatalytic oxidation (UV)	91.0	(2)
TiO_2/biochar	reed straw	300	sulfamethoxazole	10	1.25	adsorption + photocatalysis	91.3	(3)

						(UV)		
TiO_2/biochar	paper sludge and wheat husks		Reactive Blue 69	20	1.50	sonocatalysis (UV)	98.1	(4)
TiO_2/biochar	macroalgae	650	Methylene Blue	5	2.00	adsorption + photocatalysis (Vis)	99.0	(5)
TiO_2/biochar	walnut shells	500	Methyl Orange	20	0.25	photocatalytic oxidation (UV)	96.9	(6)
TiO_2/biochar	Salvinia molesta	350	Acid Orange 7	20	0.10	photocatalysis (UV)	90.0	(7)
TiO_2/biochar	raw corn cob	550	Cd(II)	50–300	1.00	adsorption	70.0	(8)
MIL-125(Ti)/biochar	Cow dung	800	TC	40	0.2	adsorption + photocatalytic (UV+Vis)	94.6	Our study

References

1. X. Peng, M. Wang, F. Hu, F. Qiu, H. Dai and Z. Cao, *Journal of Alloys and Compounds*, 2019, **770**, 1055-1063.
2. J. R. Kim and E. Kan, *J Environ Manage*, 2016, **180**, 94-101.
3. H. Zhang, Z. Wang, R. Li, J. Guo, Y. Li, J. Zhu and X. Xie, *Chemosphere*, 2017, **185**, 351-360.
4. T. Fazal, A. Razzaq, F. Javed, A. Hafeez, N. Rashid, U. S. Amjad, M. S. Ur Rehman, A. Faisal and F. Rehman, *J Hazard Mater*, 2020, **390**, 121623.
5. A. Khataee, B. Kayan, P. Gholami, D. Kalderis and S. Akay, *Ultrason Sonochem*, 2017, **39**, 120-128.
6. L. Lu, R. Shan, Y. Shi, S. Wang and H. Yuan, *Chemosphere*, 2019, **222**, 391-398.
7. S. Silvestri, M. G. Gonçalves, P. A. da Silva Veiga, T. T. d. S. Matos, P. Peralta-Zamora and A. S. Mangrich, *Journal of Environmental Chemical Engineering*, 2019, **7**.
8. M. Luo, H. Lin, Y. He, B. Li, Y. Dong and L. Wang, *Bioresour Technol*, 2019, **284**, 333-339.
